# Vicryl Reaction Following Arthroscopic Surgery: A Case Report

**DOI:** 10.7759/cureus.65255

**Published:** 2024-07-24

**Authors:** Ahmet Firat Berkay, Mehmet Ozbey Buyukkuscu

**Affiliations:** 1 Department of Orthopaedics and Traumatology, Baltalimani Metin Sabanci Bone Diseases Training and Research Hospital, Istanbul, TUR

**Keywords:** arthroscopy, polyglactin 910, “hypersensitivity”, reaction, allergic reaction, arthroscopic knee surgery, arthroscopic acl reconstruction, hypersensitivity reactions, vicryl, anterior cruciate ligament injuries and meniscus injuries

## Abstract

Reactions to Vicryl have rarely been reported in the literature. Moreover, a reaction that begins less than 24 hours postoperatively is even rarer. We present a case of Vicryl hypersensitivity that developed within 24 hours at the site of the wounds following arthroscopic anterior cruciate ligament (ACL) reconstruction and meniscal repair. A 32-year-old male patient underwent arthroscopic ACL reconstruction and lateral meniscus repair due to an injury on the soccer field. On postoperative day one, erythema and edema were observed around the wounds, and the patient complained of itching and tenderness in the same areas. Despite local and systemic medication for infection, contact dermatitis, and antiseptic allergy, no improvement was noted. Vicryl hypersensitivity was suspected. Vicryl was removed, and a polydioxanone suture (PDS) was used instead, resulting in clinical improvement for the patient. In patients presenting with erythema, edema, blistering, itching, and tenderness around the wound, Vicryl hypersensitivity as a differential diagnosis can be considered after ruling out common causes. Hypersensitivity tests may be performed in suspected cases.

## Introduction

Reactions to Vicryl (Ethicon, Bridgewater, New Jersey) have rarely been reported in the literature and are usually presented as case reports and case series. This condition can be misdiagnosed as infection, dermatitis, or allergy to antiseptics or dressing materials, resulting in prolonged, inappropriate treatment protocols and persistent symptoms.

In the literature, this reaction has been identified as a foreign body reaction. A foreign body reaction is the last stage of the inflammatory reaction of the immune system against biomaterials and implants. Macrophages play a major role in this reaction. Foreign body reactions involve the following stages: protein adsorption, acute inflammation, chronic inflammation, foreign body giant cell formation, and fibrosis. Giant cell formation is generally observed at the end of the second week [1].

Martin-Casals and Scott [2] and Demir et al. [3] reported Vicryl reactions after strabismus surgery. Sayegh et al. [4] reported three cases, and Pierannunzii et al. [5] reported two cases of Vicryl reaction following total hip arthroplasty. In this study, we report the case of a patient who developed Vicryl hypersensitivity at the wound site after arthroscopic anterior cruciate ligament (ACL) reconstruction and meniscus repair. The symptoms and findings in our patient started less than 24 hours postoperatively.

## Case presentation

The patient was informed that the data from the case would be submitted for publication and provided consent.

A 32-year-old male patient was scheduled for arthroscopic ACL reconstruction and meniscus repair approximately six months after an ACL rupture and lateral meniscus longitudinal tear caused by a soccer injury. During the procedure, three 1 cm incisions were made for the anterolateral, anteromedial, and far medial portals. Additionally, a 2 cm incision was made at the lateral side of the joint line for inside-out suture, and a 6 cm oblique incision was made on the medial side of the tibial tuberosity. An arthroscopic examination revealed that the ACL was completely torn. Through an oblique incision, the semitendinosus and gracilis tendons were harvested. ACL reconstruction was performed using the anteromedial portal technique, and the longitudinal tear in the lateral meniscus was repaired using all-inside and inside-out meniscal sutures. Vicryl was used for the closure of subcutaneous tissues in a 2 cm lateral knee incision for the inside-out suture and a 6 cm incision on the medial of the tibial tuberosity, while Prolene (Ethicon, Bridgewater, New Jersey) sutures were used for skin closure on all incisions.

**Figure 1:**
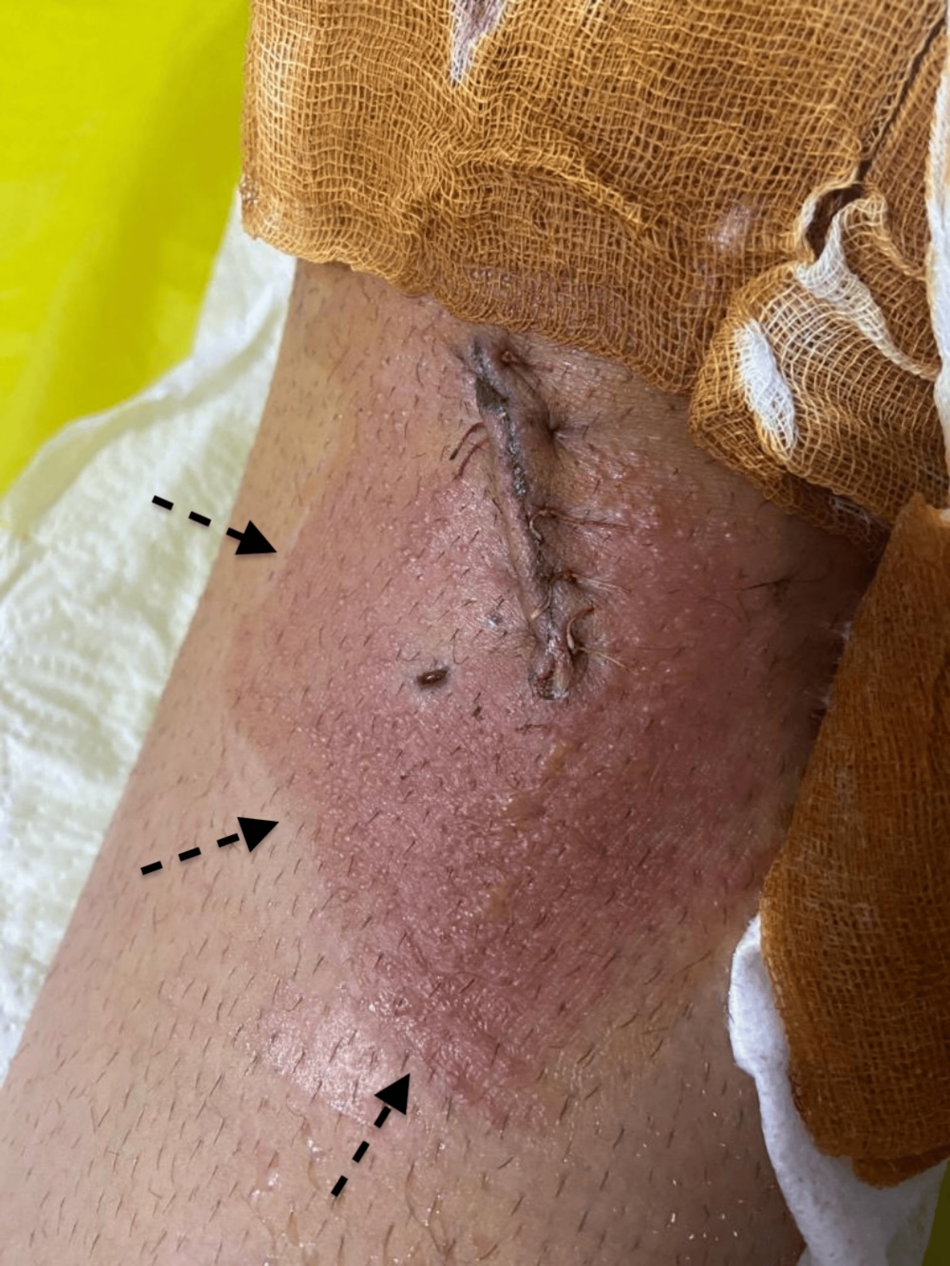
The medial of tibial tuberosity on postoperative day one Erythema and edema were visible around the oblique incision.

**Figure 2:**
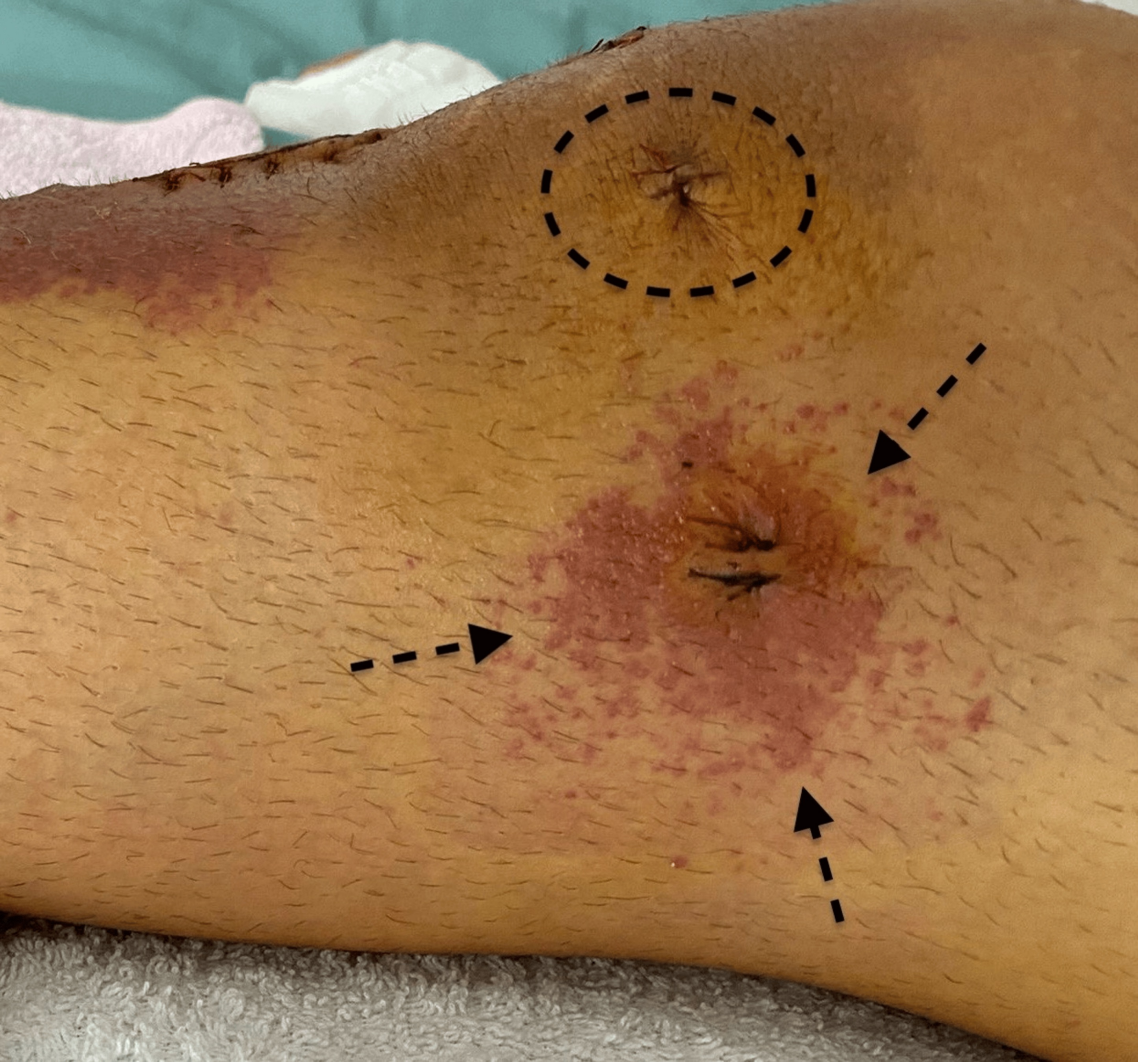
Lateral side of the knee on postoperative day one Vicryl was also used in the subcutaneous layers of the incision opened for tying the knots of the inside-out meniscus suture. Erythema was also visible on this incision (arrows). However, no reaction was detected for the portal incisions in which Vicryl was not used (circle).

On postoperative day one, erythema and edema were observed at the oblique incision for graft harvest and the lateral incision during dressing change (Figures 1, 2). The patient complained of itching and tenderness around the wounds. Initially suspected to be an allergic reaction to povidone-iodine, the antiseptic agent was switched to chlorhexidine.

**Figure 3:**
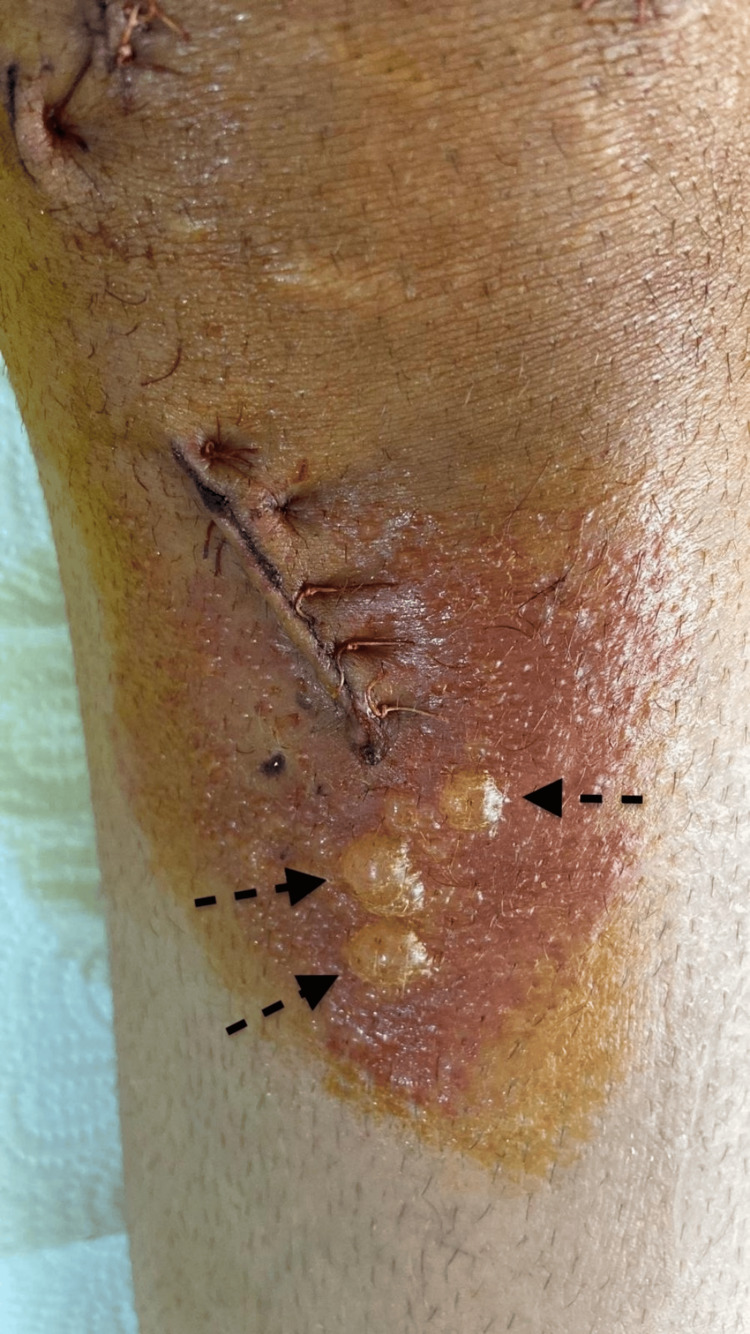
Postoperative day four Bullae were observed on the skin in the reactive areas.

On the fourth day, the patient's white blood cell (WBC) count was 9700/µL, C-reactive protein (CRP) was 1.24 mg/dL, and erythrocyte sedimentation rate (ESR) was 10 mm/hour. Bullae were observed on the skin in the reactive areas (Figure 3). Since there was no reaction observed in the other portal wounds dressed with povidone-iodine, and because of the persistence of the reaction despite using chlorhexidine, a dermatology consultation was made.

The dermatologist suspected contact dermatitis and recommended methylprednisolone 20 mg orally once daily (tapering off within a week), aluminum acetate 1% solution applied locally for 10 minutes three times a day, and Fucicort cream twice daily, and leaving the wound open applying 0.2% Eau de Goulard solution.

**Figure 4:**
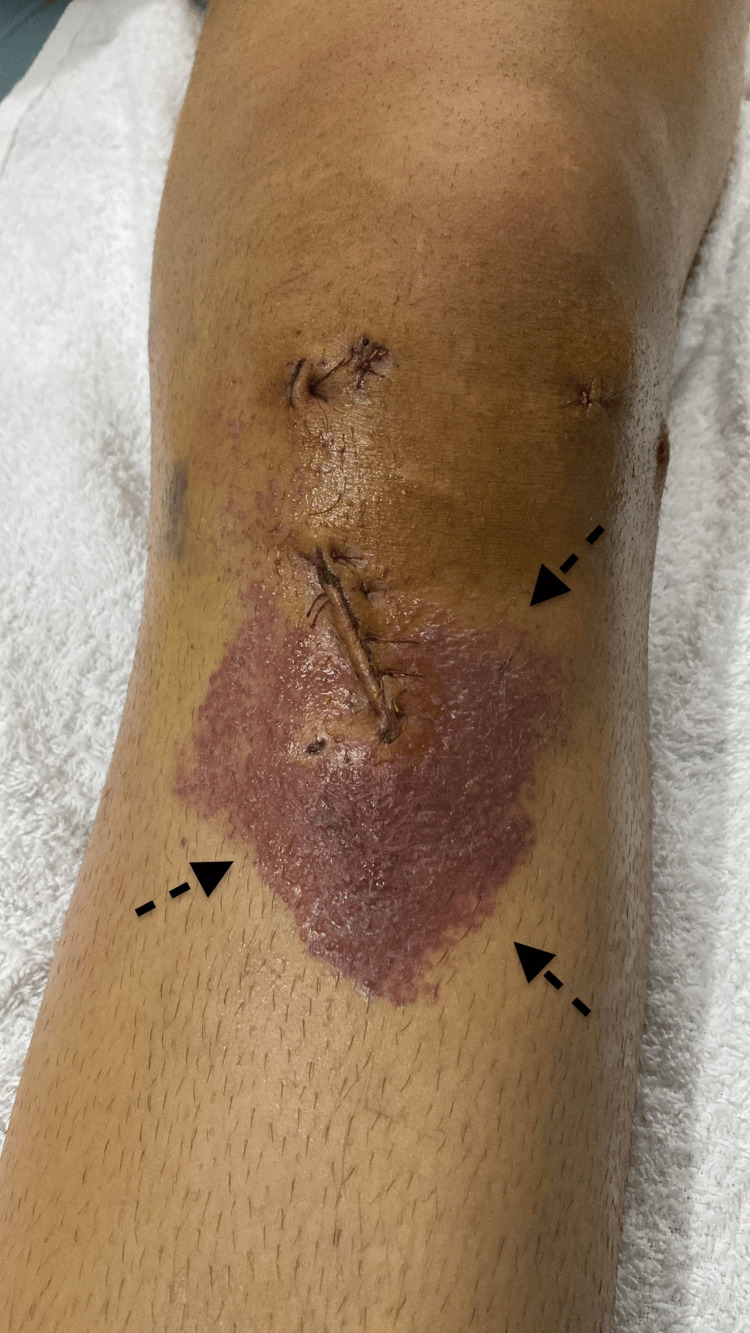
Five days after Vicryl removal Bullae and other findings began to resolve gradually after the removal of Vicryl sutures.

Following a three-day course of dermatological treatment, there was no visible improvement. However, due to the localized reaction, specifically in the areas where Vicryl was applied, Vicryl hypersensitivity was suspected. No reaction was observed at the portal incisions. Superficial debridement was performed in the operating room, and Vicryl sutures were removed and replaced with PDS (polydioxanone suture). The reactions began to subside on the following day. After three days, CRP was 0.37 mg/dL, ESR was 4 mm/hour, and WBC count was 12680/µL. The bullae almost recovered after five days (Figure 4).

**Figure 5:**
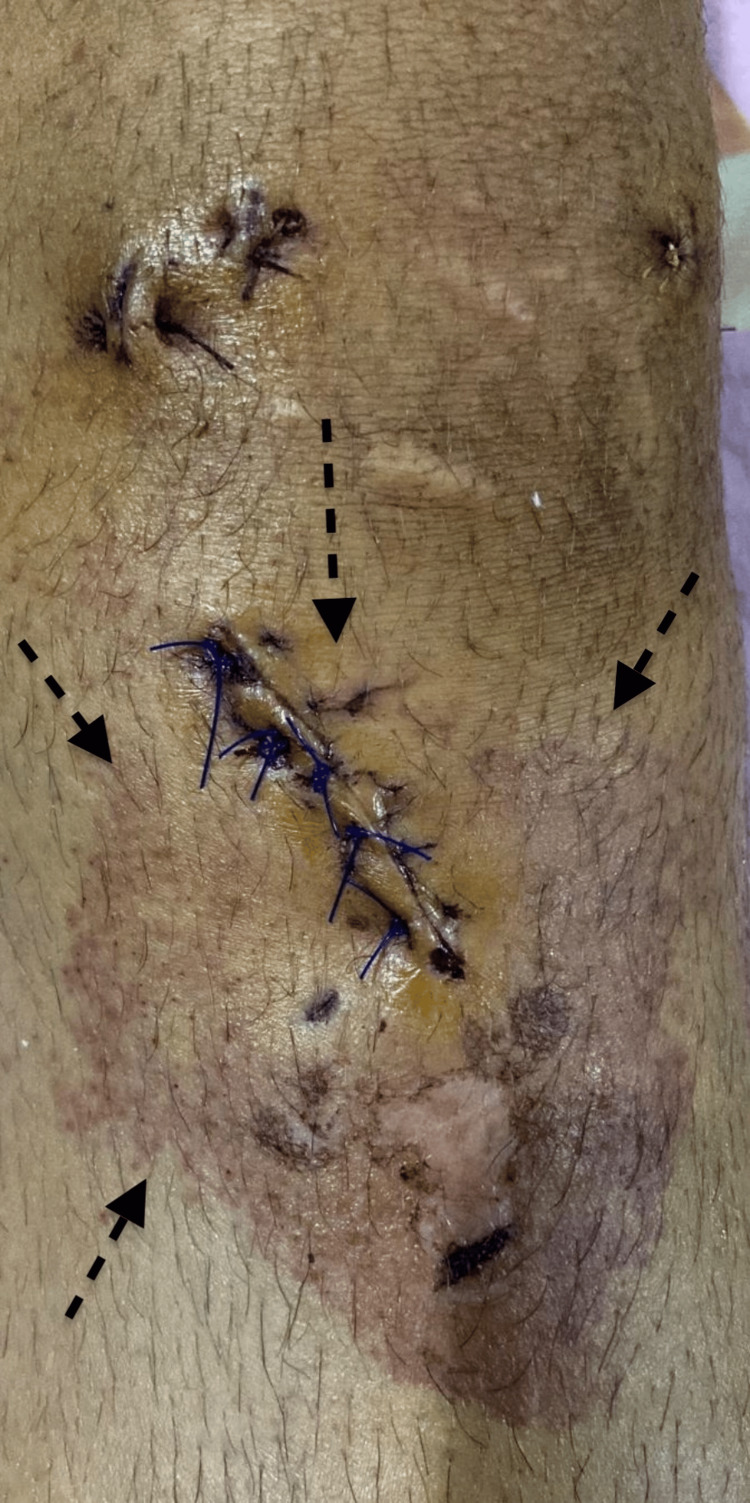
Nine days after Vicryl removal Erythema significantly regressed.

Erythema regressed significantly after nine days without any further medication (Figure 5). Sutures were removed at the end of the second week.

## Discussion

Polyglactin 910, commonly known as Vicryl, is a frequently used absorbable suture for closing both deep and superficial surgical wounds, known for causing very low tissue reactivity. Reactions to Vicryl are very rare and have been reported as case reports in the literature [2-6].

Martin-Casals and Scott documented one of the earliest case reports of a four-year-old girl who underwent strabismus surgery. They used polyglactin 910 (6-0) for muscle attachment and conjunctival closure. On the 17th postoperative day, the patient reported experiencing inflammation and discomfort lasting approximately a week. Their examination revealed hyperemia and subconjunctival swelling in the area where polyglactin 910 was used. The authors also mentioned that the complaint resolved after using topical corticosteroids [2].

Demir et al. reported another case that underwent strabismus surgery. A seven-year-old boy was operated on using a 6-0 Vicryl Rapide. The patient was discharged with topical 0.3% Ofloxacin ointment and 1% topical prednisone. However, the patient was readmitted to the hospital with conjunctival hyperemia, stinging, and photophobia complaints three days after discharge. Conjunctival cultures were negative, and the patient was unresponsive to topical corticosteroid therapy and topical 0.5% moxifloxacin therapy. Because of this situation, they also suspected of a foreign body reaction to Vicryl Rapide sutures and removed them. The patients were prescribed topical antibiotics and steroids. However, the symptoms persisted with minimal improvement. Two weeks later, 0.05% cyclosporine A ophthalmic solution was added to the therapy. The symptoms improved significantly in seven weeks, but not completely. At follow-up, the patient recovered completely after five weeks of cyclosporine A use [3].

In 2003, Sayegh et al. reported three cases of Vicryl reaction after total hip arthroplasty. Postoperatively, all patients were normal. Complaints of pain, tenderness, and erythema started in one patient in the sixth week, in another patient in the eighth week, and in another patient in the ninth week [4].

In 2015, Pierannunzii et al. reported two cases of Vicryl reaction following total hip arthroplasty. Their CRP levels dropped within normal values in two weeks. Their first case was uneventful until the ninth week and the second until the eighth week. Subsequently, the symptoms began, and they reported mildly elevated CRP levels, erythrocyte sedimentation rate, and normal WBC levels. They also reported fistulas and purulent discharge in both cases. At their debridement surgery, they encountered massive access at the deep subcutaneous level. On histological examination, what they saw were foreign body reactions in the materials collected from both of the patients [5].

Neyaz et al. reported a six-year-old patient who had a bilateral sub-tenon abscess following strabismus surgery. The preoperative and intraoperative cultures were negative. Histopathology revealed giant cells resembling foreign bodies. After the removal of Vicryl sutures, the symptoms subsided [6].

During the postoperative follow-up of our patient, we observed this reaction at the wound sites (graft harvest site and lateral incision) where Vicryl was used. The existing Vicryl materials were removed and replaced with PDS, and the patient’s symptoms and findings subsided within 10 days.

Our goal is to contribute to the literature by highlighting the possibility of this very rare condition, and this awareness may also prevent unnecessary prescription of antibiotics and steroids to patients.

## Conclusions

Postoperative inflammation at the wound site should be carefully evaluated. In patients presenting with erythema, blistering, itching, and tenderness around the wound, Vicryl hypersensitivity as a differential diagnosis can be considered after ruling out infection, contact dermatitis, and antiseptic allergy. Hypersensitivity tests may be performed in suspected cases.
